# Heart failure guidelines and prescribing in primary care across Europe

**DOI:** 10.1186/1472-6963-5-57

**Published:** 2005-08-30

**Authors:** Heidrun B Sturm, Wiek H van Gilst, Karl Swedberg, FD Richard Hobbs, Flora M Haaijer-Ruskamp

**Affiliations:** 1 Department of Clinical Pharmacology, University Medical Center Groningen, Antonius Deusinglaan 1, 9713 AV Groningen, The Netherlands; 2 Göteborg University, Department of Medicine, Sahlgrenska University Hospital, SE-416 85 Göteborg, Sweden; 3 Department of Primary Care and General Practice, Medical School, University of Birmingham, Birmingham, B15 2TT UK

## Abstract

**Background:**

Major international differences in heart failure treatment have been repeatedly described, but the reasons for these differences remain unclear. National guideline recommendations might be a relevant factor. This study, therefore, explored variation of heart failure guideline recommendations in Europe.

**Methods:**

Treatment recommendations of 14 national guidelines published after 1994 were analyzed in relation to the heart failure treatment guideline of the European Society of Cardiology. To test potential relations between recommendations and prescribing, national prescribing patterns as obtained by a European study in primary care (IMPROVEMENT-HF) were related to selected recommendations in those countries.

**Results:**

Besides the 14 national guidelines used by primary care physicians in the countries contacted, the European guideline was used in four countries, and separate guidelines for specialists and primary care were available in another four countries. Two countries indicated that no guideline was used up to 2000. Comprehensiveness of the guidelines varied with respect to length, literature included and evidence ratings. Relevant differences in treatment recommendations were seen only in drug classes where evidence had changed recently (β-blockers and spironolactone). The relation between recommendation and prescribing for selected recommendations was inconsistent among countries.

**Conclusion:**

Differences in guideline recommendations are not sufficient to explain variation of prescribing among countries, thus other factors must be considered.

## Background

Chronic heart failure is a common disease in all developed countries, and its prevalence is likely to increase further due to aging societies and improvement in therapies [1]. Therefore, the interest in good quality of heart failure management is twofold: optimal medical outcomes as well as the efficient use of resources.

Despite internationally available identical evidence, differences in heart failure therapy among countries have been repeatedly described for inpatient as well as outpatient care [2-4]. In the European Improvement of Heart Failure survey (IMPROVEMENT-HF) for instance, β-blocker-use in primary care ranged from 10% in Turkey, to 26% in the UK and 50% or more in Sweden and Hungary. ACE-use ranged from almost 50% to 75%. Since available evidence is translated into national guidelines in most countries, national recommendations may differ and accordingly contribute to such variation.

Differences among guidelines have been shown for various diseases. While actual content varied little, the major variation was found in the method and rigor of guideline development as well as the comprehensiveness [5-8]. The focus of this study is on content of published guidelines in Europe. Firstly we assess the variation in recommendations for heart failure treatment; secondly we explore their association with prescribing patterns in European countries as observed in the IMPROVEMENT-HF survey.

## Methods

Only guidelines from European countries (Belgium, Czech Republic, France, Germany, Great Britain, Hungary, Italy, Portugal, Russia, Scotland, Spain, Sweden, Switzerland, The Netherlands, Turkey) were considered. Guidelines were compared to the treatment guideline of the European Society of Cardiology (ESC) [9]. The version of 1997 was chosen as the reference guideline in order to relate recommendations to prescribing data from the European IMPROVEMENT-HF survey, which was conducted in 1999/2000 [3]. This study reported prescribing data from 11062 patients treated by 2327 primary care physicians in 14 countries.

Guidelines and recommendations for heart failure were collected through national experts (members of the ESC working group on heart failure or a recommended substitute) and by search of websites of national cardiology societies as well as Medline. Only guidelines produced by national organizations or published on a national level between 1994 and 2002 were included. Regional guidelines were not taken into account. For the main analysis the original version was always used, even if updates became available.

Two approaches were used to determine the content of national guidelines: Firstly, national experts completed a questionnaire about the content of the most commonly used guideline by primary care physicians (PCPs) in their country at the time of the IMPROVEMENT-HF study (up to 2000). Secondly, the researcher together with a native speaker with medical background used the original guideline documents to complete the questionnaire. In case of discordance, the national expert was contacted again.

As a proxy for quality, four dimensions of formal appraisal instruments [10,6] were used (provided information about authors, specified target group, information about used evidence (citations), grading of evidence).

Form and content of guidelines with different publication years or target groups were compared. Information about publication, dissemination and use of all existing national heart failure treatment guideline(s) was retrieved from the questionnaire and described.

Obtained recommendations were compared to the ESC recommendations and grouped into one of the following three categories: recommendations identical with the ESC recommendation (identical); recommendations differing from ESC (disagreement); and ESC recommendations not included or mentioned in the particular guideline (not specified). In case of disagreement specification was asked.

To relate recommendations to prescribing only recommendations of guidelines available before the survey were used. If more than one guideline was published in a country, the one mostly used by GPs (Italy, Germany and NL) was included. Prescribing data from the IMPROVEMENT-HF survey was used. Prescribing between groups of countries with identical recommendations was compared and the effect of recommendations on prescribing was analyzed using logistical regression (SPSS 11.0). Multivariate analysis was performed to account for patient characteristics influencing prescribing (age, sex and severity of disease (NYHA); significant at the 5% level in univariate analysis).

## Results

From 16 heart failure experts contacted, 13 replied citing 16 guidelines. Besides the European reference guideline, a further 14 guidelines for treatment of heart failure were included in the study (Table 1). The Scottish and the Russian documents were retrieved from the Internet and no expert opinion was available. Belgium and Turkey reported that no CHF-guideline had been used in their country. In four countries (Hungary, Portugal, Switzerland, UK) the ESC guideline was used exclusively. More than one guideline had been published in four countries, different for specialists and primary care. According to expert information, guidelines were disseminated via publication in professional literature (14 of 16), by scientific meetings (14 of 16) and Continuing Medical Education (CME; 11 of 16). Three guidelines have been updated along with the changes to the ESC guideline.

**Table 1:** List and characteristics of analyzed guidelines for heart failure

**Country**	**Pub year**	**Authors**	**Dev.**	**Target group**	**No. Ref**	**Grading**	**Epidem**	**Etiology**	**Econ. relevance**
NL	1994	CBO	+	Specialists	0	-	+	+	+
NL	1995	NHG	+	GP	191	+	+	+	-
France	1996	Individual experts	-	Specialists	129	+	+	+	+
Hungary Portugal CH, UK	1997	Task Force of European Society of Cardiology	+	All physicians*	69	+	+	+	+
Italy	1997	Task force in collaboration with society of non-hospital-cardiologists, SIC	+	Specialists	120	+	+	+	-
CZ	1998	Czech Society of Cardiology		GP	30	-	+	+	-
D	1998	Medical Commission of the physician associations (AkdA)	+	GP	66	+	+	+	-
D	1998	German Society of Cardiology, DGK	-	Specialists	213	+	+	+	-
Italy	1998	ANMCO (Cardiol. Society) and SIMG (GP-society)	-	GP	0	-	-	-	-
Scotland	1998	SIGN (Scottish Intercollegiate Guideline Network)	+	Not specified	211	+	+	+	+
Sweden	1998	SOS (National Board of Health and Welfare)	+	All physicians	0	-	+	+	-
Spain	1999	Working Group for HF of the Spanish Soc. of Cardiology	+	GP and specialists	113	+	+	+	+
Sweden	2000	MPA (Medical Product Agency)		Physicians other than cardiologists	0	+	+	+	+
Russia	2001	Ju. N. Belenkow, Scientific Research Institute of Cardiology, Ministry of Health, Moscou	-	Not specified	0	-	+	+	+
CH	2002	Working group on HF of the Swiss society of cardiology	+	Not specified	24	+	+	+	+

Total (n = 15)			9	9	10	10	13	13	8

+ included, - not included, GP = General Practitioner, NL: The Netherlands, CZ: Czech Republic, CH: Switzerland, D: Germany, Swe: Sweden

**Dev.**: method of development is outlined in the document. **Target group**: as specified in the GL, according to publication or as indicated by specialist in questionnaire.

**Grading: **levels of scientific evidence given with recommendations. "Quality" included specification of development, target group, evidence levels and literature.

* In Portugal the ESC-guideline was targeted at specialists. # This version was last updated 11/2001

### Form

Presentation and comprehensiveness of the guidelines varied (Table 1). Length along with included literature, ranged from comprehensive publications to handouts comprising only flowcharts. The method of development was clearly stated in 8, evidence ratings were included in 10 guidelines. National specialist societies or physician organizations were the authors in all but two cases. Epidemiology was discussed in all guidelines, etiology in all but the Italian leaflet for general practitioners. The economic relevance of heart failure was mentioned in about half of the documents. In 10 cases a target group of physicians was specified in the guidelines or was defined by the publishing organization or journal, which addressed specific groups of physicians. All guidelines specifically directed at specialists (except the oldest, the Dutch CBO) included more than 100 citations and gave evidence levels for their recommendations, whereas, on the other hand, guidelines without evidence levels or less than 30 citations were directed at PCPs or did not specify the target group (Table 1). Guidelines for specialists contained more detailed recommendations than the others (10% non-specified recommendations in guidelines for specialists as opposed to 24% in the others, Table 2), yet the content of included recommendation did not differ vastly. Overall, three of the 15 guidelines provided information on all four quality dimensions, six did not specify either the target group or the development procedure but all the remaining criteria.

**Table 2:** Overall differences in content between guidelines for different target groups

	recommendations*	Identical with ESC [%]	Disagreement [%]	Not specified [%]
Guidelines for†	Specialists (n = 5)	70	19	10
	other (n = 9)	63	13	24
	All (n = 14)	65	16	19

*Each guideline was checked for 16 recommendations and compared to the European guideline (ESC) of 1997

† see Figure 1.

### Content

#### Similarities

**Figure 1:**
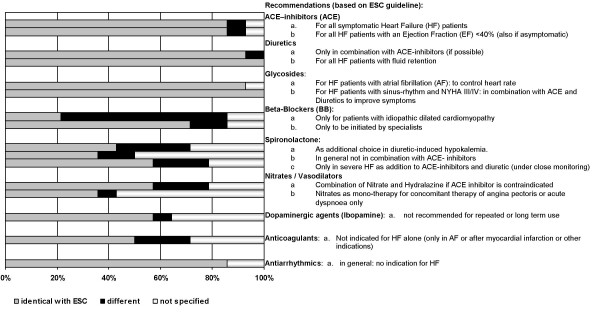
**Variation in recommendations included in European guidelines**. Recommendations relating to one drug are grouped; corresponding results (percentages of identical, different or not specified recommendations) are shown in adjacent horizontal bars. n = 14 national guidelines (other than the European Society of Cardiology (ESC)-guideline).

Drug therapy recommendations for diuretics, ACE-inhibitors and glycosides did not reveal major differences (Figure 1). Only the Dutch NHG-guideline for primary care recommended diuretics as first drug choice prior to ACE inhibitors. The cut-off value for the ejection fraction below which ACE-treatment should be initiated varied between 40% (ESC) to 25% (Russia).

#### Differences

The indications for β-blocker use varied (Figure 1). Whereas 9 guidelines recommended their use for all stable CHF patients (three excluded most severe cases, NYHA class IV), the ESC guideline along with three others advocated β-blockers only for idiopathic dilated cardiomyopathy. Those were published in 1994, 1998 and 2000.

The recommendation to restrict their initiation and control to specialists was included by the majority, with an overall range from: not to be used by GPs at all (Dutch NHG 1994) to: by any doctor (Spain, 2000). The relation to publication time was generally consistent: The limitation to specialists was included mainly in earlier guidelines and the two guidelines clearly disagreeing were both from 2000. Yet, in Switzerland the limitation was still included in 2002.

Also recommendations for spironolactone showed differences (Figure 1): The Dutch CBO (1994) and the Italian brochure (1997) did not mention spironolactone at all. Before 1999 agreement about the use of spironolactone as an additional diuretic in hypokalemia was more frequent (5 of 10 vs. 1 of 4 after 1999). Also more frequent was the recommendation not to combine spironolactone with ACE. After 1999 this warning was replaced by the recommendation to use spironolactone in addition to ACE and other therapy in severe heart failure.

Only minor variation was found in recommendations for other drug groups: The combination of nitrates and hydralazine as additional symptomatic treatment choice was only recommended by the two German guidelines. Of the 9 guidelines addressing anticoagulation, three specified their indication for patients with an EF < 20–25% (D, Italy) or NYHA 3 and 4 (Russia).

### Updates in guidelines

When comparing the different versions of the German, the Dutch CBO and the ESC guideline, the emergence of new evidence for β-blockers and spironolactone is reflected by the order in which it is discussed in the guidelines: β-blockers in the old versions were the fourth or later topic, in the new versions they were moved to second or third. Spironolactone was no longer discussed within the diuretics-section but separately. For other drug groups no major changes were found. In the Dutch document the use of tests and particularly echocardiography to establish diagnosis was more clearly recommended.

### Guidelines and prescribing

Recommendations for ACE-inhibitors did not differ between the countries, yet prescribing ranged from 48% in Sweden and 76% in Hungary[3]. Also glycoside-use ranged from 24% in the UK to 45% in Italy and 55% in Turkey.

Where major differences in recommendations were present (for spironolactone and β-blockers), the relation to prescribing was inconsistent. Where recommendations warned about the combination of spironolactone with ACE (before 2000, average year of publication: 1997) this drug-combination was prescribed significantly less than where no warning was included in the guideline (Table 3).

**Table 3:** Actual prescribing in relation to recommendations

**Recommendation**	**Countries ** (% of total)	**Year of publication ** Range (mean)	(%)	**Drug use ** % patients with OR (95%CI)	
**Spironolactone **in general not to be given in combination with ACE			**ACE + spironolactone**		
			
				univariate	multivariate
			
All countries*	10381 (100%)		6.6		
				
Warning (Identical)	NL, Cz, F, D, Swe (36,5%)	1995–1998 (1997)	5,7	0.78 (0.66–0.91)	0.74 (0.63–.87)
				
no warning/no recommendation^†^	Remaining (63,5%)	1998 (1998)	7,2	1	1

**β-blockers **are indicated only for patients with IDCM (idiopathic dilated cardiomyopathy)			**β-blocker**		
			
All countries*	10381 (100%)		33.8		
Restricted indication (identical)	Cz, H, CH, UK (28.6%)	1998 (1998)	40.4	1	1
No restriction	Remaining^† ^ (71.4%)	1995–1998 (1997)	31.1	0.67 (0.61–0.73)	0.69 (0.63–0.76)

*Poland was excluded from the analysis, because no GL information was available.

^†^includes all countries without guideline before 2000 and guidelines without recommendation. In the case of NL and Italy, the specific guideline for GPs has been used.

In contrast, β-blockers were prescribed more likely if recommendations restricted their indication to the subgroup of patients with idiopathic dilated cardiomyopathy (Table 3). Those guidelines (available before 2000) were all published 1998 with the exception of the Dutch guidelines for specialists from 1994; the ones without specification were published between 1995 and 1998. Also if the Dutch and Italian guidelines for specialists were used in the statistical model, the same trend was found. In both cases correcting for patient characteristics did not change the pattern either.

## Discussion

The goal of this study was to assess the degree of variation between recommendations for heart failure treatment in Europe as an potential factor explaining international variation in prescribing. To our knowledge the content of a wide range of European heart failure guidelines and its relation to actual prescribing data has not been assessed before [5].

Similar to other studies comparing guideline recommendations [8,6] our results did not reveal major differences. With the exception of one of the oldest, all guidelines recommended ACE-inhibitors as first line medication. Recommendations for diuretics and glycosides were basically identical.

Most variance was found where the emergence of new evidence induced changes in established therapy during the study period, namely for spironolactone [11] and β-blockers [12]. The role of spironolactone in severe heart failure was changed by the RALES study in 1999. This is reflected in a switch from warning about the combination of spironolactone with ACE inhibitors in earlier guidelines to recommending this combination in the later ones. The growing importance of β-blockers for CHF therapy was reflected in a trend to widen the indication and not to restrict its prescribing to specialists any more. However, the respective conservative recommendation was still included in one newer guideline (from 2000 or 2002). On the other hand different recommendations were not reflected in the average publication year of guidelines. Thus, new evidence is not always taken up at the same pace; some countries appear to be more conservative or cautious than others.

Still, as to be expected the frequency of updates influenced the time lag between emergence and inclusion of new evidence. The two German guidelines were updated within three years and new evidence was reflected in the changes made. In the Netherlands the guideline for primary care from 1995 was updated together with the 1994 guideline for specialists in an interdisciplinary manner only in 2002. Thus, despite being early in guideline development, this time span between updates resulted in some recommendations, which were not in accordance with newer evidence for some years.

The target group to which a guideline is directed appeared to play a role for both, the formal scientific appearance and the content. Guidelines for primary care physicians included fewer citations, gave evidence levels less frequently, and included fewer specific recommendations. It has been shown, that form and credibility of the guidelines' authors influence their uptake [13,14]. Given the fact, that primary care is where prescribing for heart failure mainly takes place in most European countries [15,16] and that primary care physicians tend to be less in accordance with newer evidence than specialists [17-20], guidelines directed at general practitioners should be given special attention. In our study, in six countries no specific guideline for primary care physicians could be detected. Thus, to adjust form and content of guidelines to primary care physicians' needs might be one way of improving the implementation of evidence in primary care.

By associating national recommendations with prescribing data we aimed to further explore the role of guidelines in international variation of heart failure therapy. The found relationship, however, appeared to be inconsistent: On the one hand, guideline-recommendations for major drug classes were similar while prescribing e.g. for ACE-inhibitors differed by about 25% among countries. On the other hand, when recommendations differed between countries, prescribing was not always correlated. Another example from our data for the weak link between prescribing and guideline recommendations is the Dutch case, where despite the conservative guideline recommendations in 1999 the frequency of ACE and β-blocker prescribing ranged at the European mean.

### Limitations to the study

In order to better understand the relationship between heart failure prescribing and national recommendations in European primary care, this study focused on published content rather than on the rigor of development of guidelines. In view of this focus guidelines were assessed in a explorative, descriptive manner. Instead of using one formal guideline appraisal instrument [21] we assessed major dimensions according to the checklists provided by Grilli [10] and Kulig [6] to give an indication about quality. In depth analysis of recommendations was limited to major drug groups and therefore minor differences might not have been considered.

Only European guidelines were included in this study, as they were to be analyzed in connection with the prescribing data. For the same reason the European guideline of 1997 (ESC) formed the basis for the content analysis rather than the later version of 2001. Nevertheless newer recommendations were included and therefore changes over time could still be detected.

In the statistical analysis, we accounted for differences in selected patient characteristics between the compared groups that are relevant determinants of prescribing, however other potentially relevant patient (or doctor) factors were not included. In addition, in this cross sectional analysis differences in time of guideline publication and emergence of new evidence could not be accounted for. Therefore prescribing data and its link to recommendation can only give an indication about general relations between the two.

Further, countries can only be grouped according to published national guidelines. This might not in all cases reflect recommendations actually used by physicians.

Detailed information about the implementation process [22-24] as a major aspect for a guideline's impact on prescribing was beyond the scope of this study, although our expert replies suggest roughly comparable approaches of guideline dissemination. The range of different forms as well as of authoring groups may have further compromised comparability of acceptance and uptake.

Although recruitment procedures of practices aimed to include comparable national populations, variations in physician characteristics were still present. Further, even though only primary care practices were included, there might have been relevant differences in national organization of care.

## Conclusion

In the majority of the European countries surveyed national heart failure treatment guidelines were available at the time of the study. Nine of 15 guidelines included information about at least three quality dimensions (specified authors and development, specified target group, citations and grading of evidence), still form and comprehensiveness varied substantially.

Differing degrees of comprehensiveness for different user groups suggest that increased attention to the target group of primary care when issuing recommendations might enhance the uptake of guidelines, bearing in mind that CHF is treated primarily in primary care.

This study suggests that national guidelines only play a minor role in explaining differences in heart failure therapy between countries. The majority of recommendations were similar, differences were found mainly within drug groups where evidence has undergone recent change. Also, in the case of distinct national recommendations, prescribing within countries does not appear to consistently follow the advice. These data emphasize the relevance of country specific factors for prescribing at a national level, one of which may be the implementation of guideline recommendations.

## Competing interests

HS, WvG, FMH-R, KS declare that they have no competing interests.

FDRH has received fees, research funding or sponsorship, on an occasional basis, from a number of pharmaceutical or diagnostics companies active in the field of heart failure.

## Authors' contributions

HS, FMH, WG participated in the study design. HS and FMH developed the questionnaire; HS collected the guidelines, followed up with experts, analyzed the documents with native speakers and drafted the manuscript. FRH, KS, WG and FMH advised which experts to contact, helped with interpretation of results and with the draft. All authors read and approved the final manuscript.

## Pre-publication history

The pre-publication history for this paper can be accessed here:



## Supplementary Material

Additional File 1 Individual recommendations per country. part a-c gives country wise results for each recommendation. Click here for file
