# Supplementary material for: Heart failure guidelines and prescribing in primary care across Europe
Source: BMC Health Serv Res. 2005 Aug 30;5:57. doi: 10.1186/1472-6963-5-57 (PMC1236923; doi:10.1186/1472-6963-5-57)
Supplement: Additional File 1 — Individual recommendations per country. part a-c gives country wise results for each recommendation. [file 1472-6963-5-57-S1.doc]

Additional table: Individual recommendations per country

*a)* Recommendations for diagnostic and counseling:

| Box a: questionnaire questions (q) (Recommendations) The following recommendation is included in the guideline:    q1: Clinical symptoms must be supplemented by more objective tests to confirm the diagnosis of HF  q2: The establishment of cardiac dysfunction is needed to confirm the diagnosis of HF. Echo is method of choice for that.  Recommended counseling:  q3: Restricted sodium intake in severe HF  q4: avoid excess fluid and alcohol intake  q5: stop smoking  q6: weight control  q7: Exercise according to severity, discourage rest |  | diagnosis | | counselling | | | | |
| --- | --- | --- | --- | --- | --- | --- | --- | --- |
| **Questions:** |  | **q1** | **q2** | **q3** | **q4** | **q5** | **q6** | **q7** |
| **NL / CBO** | 1994 | -****** | + | + | + | . | + | + |
| **NL / NHG** | 1995 | + | -^#^ | + | + | . | + | + |
| **France** | 1996 | . | . | + | + | . | + | + |
| **Italy/ SIC** | 1997 | + | + | + | + | + | + | + |
| **Czech Rep** | 1998 | + | + | + | + | + | + | + |
| **D / AkdA** | 1998 | + | + | + | + | + | + | + |
| **D / cardiol. Society** | 1998 | + | + | + | + | + | + | + |
| **Italy/ SIMG** | 1998 | . | + | + | + | + | + | + |
| **Scotland** | 1998 | + | + | + | + | + | + | + |
| **Sweden / Sos** | 1998 | + | + | + | + | + | + | + |
| **Sweden / MPA** | 1999 | + | + | + | + | + | + | + |
| **Spain** | 2000 | + | + | . | . | . | . | . |
| **Russia** | 2001 | . | . | + | + | . | + | + |
| **Switzerland** | 2002 | + | + | . | + | . | + | + |
| **N=14** |  |  |  |  |  |  |  |  |
| **Identical (+)@** |  | 10 | 11 | 12 | 13 | 8 | 13 | 13 |
| **Disagree (-)** |  | 1 | 1 | 0 | 0 | 0 | 0 | 0 |
| **Not specified (.)** |  | 3 | 2 | 2 | 1 | 6 | 1 | 1 |

*b)* Recommendations for ACE-inhibitors, diuretics, spironolactone and ß-blockers

| **Box b:**  The following recommendation is included in the guideline: ACE-inhibitors q8: For all symptomatic HF-patients  q9: For all HF-patients with an Ejection Fraction (EF) <40% (also if asymptomatic)  **Diuretics**  q10: Only in combination with ACE-inhibitors (if possible)  q11: For all HF-patients with fluid retention  **Spironolactone:**  q12: As additional choice in diuretic-induced hypokalemia.  q13: In general not in combination with ACE- inhibitors  q14: Only in severe HF as addition to ACE-inhibitors and diuretic (under close monitoring)  **Beta-Blockers:**  q15: Only for patients with idiopathic dilated cardiomyopathy  q16: Only to be initiated by specialists |  | ACE | | Diuretics | | Spironolactone | | | ß-Blocker | |
| --- | --- | --- | --- | --- | --- | --- | --- | --- | --- | --- |
|  |  | **q8** | **q9** | **q10** | **q11** | **q12** | **q13** | **q14** | **q15** | **q16** |
|  |  |  |  |  |  |  |  |  |  |  |
| **NL / CBO** | 1994 | + | + | + | + | . | . | . | + | + |
| **NL / NHG** | 1995 | - | - | - | + | . | + | - | . | + |
| **France** | 1996 | + | + | + | + | + | + | + | - | + |
| **Italy/ SIC** | 1997 | + | + | + | + | - | . | - | - | + |
| **Czech Rep** | 1998 | + | + | + | + | - | + | - | + | + |
| **D / AkdA** | 1998 | + | + | + | + | + | . | + | - | + |
| **D / DGK** | 1998 | + | + | + | + | + | + | + | - | + |
| **Italy/ SIMG** | 1998 | + | . | + | + | . | . | . | . | . |
| **Scotland** | 1998 | + | + | + | + | + | . | + | - | + |
| **Sweden / Sos** | 1998 | + | + | + | + | + | + | + | - | + |
| **Spain** | 2000 | . | + | + | + | - | - | + | + | - |
| **Swed / MPA** | 2000 | + | + | + | + | + | - | + | - | - |
| **Russia** | 2001 | + | + | + | + | - | . | . | - | . |
| **Switzerland** | 2002 | + | + | + | + | . | . | + | - | + |
| **N=14** |  |  |  |  |  |  |  |  |  |  |
| **Identical (+)** |  | 12 | 12 | 13 | 14 | 6 | 5 | 8 | 3 | 10 |
| **Disagree (-)** |  | 1 | 1 | 1 | 0 | 4 | 2 | 3 | 9 | 2 |
| **Not specified (.)** |  | 1 | 1 | 0 | 0 | 4 | 7 | 3 | 2 | 2 |

*c)* Recommendations for glycosides, nitrates, dopaminergic agents, anticoagulants and anti-arrhythmics

| Box c Included recommendation:  **Glycosides**:  q17: For HF patients with atrial fibrillation: to control heart rate  q18: For HF patients with sinus-rhythm and NYHA III/IV: in combination with ACE-inhibitors and Diuretics to improve symptoms  Nitrates / Vasodilators  q19: Combination of Nitrate and Hydralazine if ACE inhibitor is contraindicated  q20: Nitrates as mono-therapy for concomitant therapy of angina pectoris or acute dyspnoea only  q21: **Dopaminergic agents (Ibopamine)**: not recommended for repeated or long term use  q22: **Anticoagulants**: Not indicated for HF alone (only in AF or after MI or other indications)  q23: **Antiarrhythmics**: in general: no indication for HF |  | Glycosides | | Nitrates | |  |  |  |
| --- | --- | --- | --- | --- | --- | --- | --- | --- |
|  |  | **q17** | **q18** | **q19** | **q20** | **q21** | **q22** | **q23** |
|  |  |  |  |  |  |  |  |  |
| **NL / CBO** | 1994 | + | + | + | . | . | . | + |
| **NL / NHG** | 1995 | + | + | + | + | + | . | + |
| **France** | 1996 | + | + | + | - | + | + | + |
| **Italy/ SIC** | 1997 | + | + | + | + | - | - | + |
| **Czech Rep** | 1998 | + | + | + | + | + | + | + |
| **D / AkdA** | 1998 | + | + | - | . | + | + | + |
| **D / DGK** | 1998 | + | + | - | . | + | - | + |
| **Italy/ SIMG** | 1998 | + | + | . | . | . | . | . |
| **Scotland** | 1998 | + | + | + | . | . | + | + |
| **Sweden / Sos** | 1998 | . | + | + | . | . | + | . |
| **Spain** | 2000 | + | + | . | . | + | + | + |
| **Swed / MPA** | 2000 | + | + | - | + | + | + | + |
| **Russia** | 2001 | + | + | . | . | . | - | + |
| **Switzerland** | 2002 | + | + | + | + | + | + | + |
| N=14 |  |  |  |  |  |  |  |  |
| **Identical (+)** |  | 13 | 14 | 8 | 5 | 8 | 7 | 12 |
| **Disagree (-)** |  | 0 | 0 | 3 | 1 | 1 | 3 | 0 |
| **Not specified (.)** |  | 1 | 0 | 3 | 8 | 5 | 4 | 2 |

*q: Question

** Recommendation: Tests are “desirable” if reasonable with respect to age and co-morbidity

^#^ Recommendation: PCP’s can’t usually initiate Echo without specialists. Its usefulness for general practice patients is not clearly established

@ See methods-section
